# Safety and effectiveness of bariatric surgery: Roux-en-y gastric bypass is superior to gastric banding in the management of morbidly obese patients: a response

**DOI:** 10.1186/1754-9493-3-17

**Published:** 2009-07-28

**Authors:** Sunil Bhoyrul, John Dixon, George Fielding, Christine Ren Fielding, Emma Patterson, Lee Grossbard, Vafa Shayani, Marc Bessler, David Voellinger, Helmuth Billy, Robert Cywes, Timothy B Ehrlich, Daniel B Jones, Brad M Watkins, Jaime Ponce, Matthew Brengman, Gregory Schroder

**Affiliations:** 1 Dept. of Surgery, Scripps Memorial Hospital, La Jolla, CA, USA; 2 Baker IDI Heart and Diabetes Institute, Melbourne, Victoria, Australia; 3 Dept. of Surgery, NYU School of Medicine, New York, NY, USA; 4 Laparoscopic Bariatric Surgery Program, Legacy Hospital, Portland, OR, USA; 5 Florida Obesity Surgical Associates, Tampa, FL, USA; 6 Bariatric Institute of Greater Chicago, Hinsdale, IL, USA; 7 Department of Surgery, Columbia University, New York NY, USA; 8 Southeast Bariatrics Charlotte, NC, USA; 9 Advanced Surgical Associates, Ventura, CA, USA; 10 Dept. of Bariatric Surgery, Memorial Hospital, Jacksonville, FL, USA; 11 Metabolic and Bariatric Surgery, St. Vincents Medical Center, Bridgeport, CT, USA; 12 Section of Minimally Invasive Surgery, Harvard Medical School, MA, USA; 13 Department of Bariatric Surgery, Synchrony Health Chicago, Oak Brook, IL USA; 14 Bariatric Surgery Dept., Hamilton Medical Center – Dalton Surgical Group, Dalton, GA, USA; 15 Richmond Surgical Group, St. Mary's Hospital, Richmond, VA, USA

## Abstract

**Background:**

The recent article by Guller, Klein, Hagen was reviewed and discussed by the authors of this response to critically analyze the validity of the conclusions, at a time when patients and providers depend on peer reviewed data to guide their health care choices. The authors of this response all have high volume bariatric surgery practices encompassing experience with both gastric bypass and gastric banding, and have made significant contributions to the peer reviewed literature. We examined the assumptions of the paper, reviewed the main articles cited, provided more evidence from articles that were included in the materials and methods of the paper, but not cited, and challenge the conclusion that Roux-en-Y gastric bypass is superior to gastric banding.

**Results and discussion:**

The paper by Guller et al was subject to significant bias. The authors did not demonstrate an understanding of gastric banding, selectively included data with unfavorable results towards gastric banding, did not provide equal critique to the literature on gastric bypass, and deliberately excluded much of the favorable data on gastric banding.

**Conclusion:**

The paper's conclusion that gastric bypass is the procedure of choice is biased, unsubstantiated, not supported by the current literature and represents a disservice to the scientific and health care community.

## Letter

We read with interest the recent article, Safety and effectiveness of bariatric surgery: Roux-en-Y Gastric Bypass is superior to gastric banding in the management of morbidly obese patients [1]. At a time when patients, health care providers, and the scientific community are faced with more choices, increasing costs, and complex decisions, there is an obligation to provide unbiased data that accurately reflects ***current ***practice standards and guides the community into asking scientifically valid questions. Regrettably, the article by Guller et al did not do this.

### Invalid and biased assumptions

The authors' assumptions that gastric banding is exclusively restrictive, and performed by general surgeons without specific training in laparoscopic and bariatric surgery, is unfounded, reflects the biased premise of the authors. High volume bariatric surgeons realize that what separates gastric banding from other "exclusively restrictive" procedures such as the sleeve gastrectomy or the vertical banded gastroplasty is adjustability. Mechanisms for restriction and malabsorption do not reflect the mechanism of action of either procedure but this is not a major issue in our response. Adjustability allows surgeons to tailor weight loss, satiety, and hunger, and provides tremendous leverage in the doctor-patient relationship. It has the potential to enhance compliance, and provide superior outcomes

### Selective inclusion of unfavorable data, and exclusion of favorable data

Although Guller et al claim to have identified all relevant literature published up to March 2009, they base most of their conclusions on a review article by Tice et al, and a follow up study by Suter and colleagues but provide no adequate description of their search method or how they selected a small number of articles to focus on [2,3]. The review article by Tice and colleagues had many self-acknowledged limitations. Tice and colleagues started off with the premise that "gastric bypass is the standard of care", and were partially funded by an insurance company that has often resisted the adoption of gastric banding in the US. Furthermore, they based their conclusions on 14 comparative studies that they acknowledged were of low quality, and also acknowledged that the median follow up time for the comparative studies was only 18 months. Most authors, including Tice and colleagues, and all the authors of this response, believe that valid conclusions comparing the efficacy of gastric bypass to gastric banding cannot be made at 18 months. Suter's long term follow up study is chosen exclusively by Guller and colleagues in their review of long term studies of gastric banding. Closer examination of the Suter paper (which is clearly an outlier from the majority of the published data on gastric banding) reveals that the majority of the procedures were performed using techniques that are no longer used (as they had recognized complications) and devices that are no longer common in the United States, and worldwide. This is the very same Swiss group that has reported almost universal nutritional deficiencies within 2 years of RYGB. Half the patients had the bands placed in a "perigastric" location. This placement is no longer used as it has been recognized as a cause of preventable slippage. The Pars Flaccida technique is now the procedure of choice in the US. Suter's patients had a variety of different bands implanted, none of which are still in use today. Larger bands, with a larger range of adjustability, are now utilized worldwide. A critical, and relatively recent development not mentioned in Suter's paper, is the widespread use of clinical pathways that emphasize frequent adjustments, comprehensive care, and objective measurement of hunger and satiety to guide band adjustments. We find it surprising that Guller et al chose a study which relied heavily on placing bands that are not commonly used, using a technique that is no longer used, and without any mention of the importance of follow up and comprehensive care, to make conclusions about the status of gastric banding in 2009 – an operation that is performed differently with different devices, and a radically different approach to post-operative care.

Guller et al failed to review the majority of available contemporary data that are highly favorable towards gastric banding. Gastric banding is clearly associated with a lower operative mortality than gastric bypass.[4]. Excellent long term data by Favretti with 12 year follow up, and an 11.9% reoperation rate, Biagini with 591 patients followed up over 10 years, and Ponce with >1000 patients followed up for 4 years would have been more appropriate representations of previous and current surgical techniques [5-7]. Short and medium term follow up studies with excellent results have been published by many authors including a 2 year study with 400 consecutive patients by Watkins et al showing 48–55% EWL at 1 year. Spivak et al. [8,9]. followed 500 patients for 1 year and produced 47% EWL. Shayani and his colleagues followed 409 patients for 3 years and demonstrated 53.3% EWL [10]. Ren and colleagues placed 749 gastric bands with 52% EWL at 3 years [11]. Only 1.5% of the bands were removed and the rate of resolution of diabetes was equivalent in patients who had a bypass and those who had a gastric band. In another paper Shayani demonstrated the feasibility of gastric banding in the super obese, a finding that was confirmed by Montgomery and colleagues [12,13]. Ponce has demonstrated excellent stewardship in the field and not only produced 63% EWL in over 1000 patients followed for 4 years, but showed that of the 40 patients who required reoperation, 95% continued to have a good result. Ponce also showed that after the surgical technique was modified from a perigastric technique to a pars flaccida technique the reoperation rate fell from 22% to 1–2%. This finding underscores the invalidity of using Suter's paper (with his outdated technique) as the basis for many of the conclusions that Guller and colleagues reached. Dixon demonstrated in a randomized clinical trial that adjustable gastric banding produced a 73% resolution rate for type 2 diabetes [14]. This finding headlines the numerous reports of resolution of obesity related comorbidities with gastric banding, the scope of which is beyond this response. Finally, two key studies demonstrate that gastric banding reduces overall mortality when compared with matched community controls. The Italian study and Australian studies reported hazard ratios of 0.36 (95% CI, 0.16 – 0.80) and 0.28 (0.10 – 0.80), respectively [15,16]. These impressive figures are enhanced by the safety of the adjustable gastric banding procedure.

### Inadequate critique of the literature on gastric bypass

We are also surprised by the paucity of citations for patients undergoing bypass in the paper by Guller. Far from a side by side comparison of the 2 procedures, they focus most of their attention citing outdated, poor quality data against gastric banding and hardly any data on gastric bypass. There is also the premise that long term data regarding gastric bypass is abundant and of high quality. For a procedure that has been performed for over 40 years, long term data is scarce and of low quality. We refer them to Ren and colleagues who have an excellent high volume academic bariatric surgery practice with large numbers of gastric bypass and gastric banding patients [17]. In their outcomes data, the overall complication rates from banding and bypass were approximately 9% and 23%, respectively, but serious complications were 0.2% for banding and 2% for bypass.

### The real questions

This paper, focuses a lot of energy on making questionable comparisons between 2 well established procedures with proven efficacies. Our feeling is that we need to spend more time understanding the science of obesity, the mechanism of action of bariatric surgery, and stratifying the care of our patients to the procedures best suited to their illness [18]. Little if any work exists on using body composition, physiological profiles, behavior analysis, and even genomics to direct patient care to the most appropriate procedure. We look forward to such useful reviews and scientific breakthroughs.

## Abbreviations

EWL: Excess Weight Loss.

## Competing interests

Sunil Bhoyrul, Clinical Research Studies funded by Allergan and Ethicon Endo Surgery.

John Dixon, Clinical Research Allergan Health, Consultant to Allergan Health, Bariatric Advantage, Scientific Intake

George Fielding, None

Christine Ren, None

Emma Patterson, None

Lee Grossbard, Proctor Allergan, Research support Synovis

Vafa Shayani, Allergan advisory board, proctor

Marc Bessler, Educational Grants: Olympus, Covidien; Ethicon fellowship support and consulting; Allergan advisory board

David Voellinger, Allergan advisory board, Proctor, Research Grant; Ethicon Endo-Surgery, Speaker's Bureau, Emmi Solutions Medical Advisory Board; USGI Medical Research Grant

Helmuth Billy, None

Robert Cywes, Allergan Advisory Board

Timothy B. Ehrlich, Educational Grant support; Allergan, Covidien, Gore; Allergan advisor

Daniel B. Jones, None

Brad M. Watkins, None

Jaime Ponce, Allergan: proctor, speaker, advisor, research support

Ethicon: proctor, speaker, consultant, research support

Matthew Brengman, None

Gregory Schroder, None

Christine Ren, Allergan Advisory Board, Speakers Bureau, Research Grants, Education    Grant. -  Ethicon Research Grant, Education Grant - Exploramed Consultant

Emma Patterson Advisory board member, proctor and research consultant (unrestricted grant) for Allergan Health  

## Authors' contributions

SB and JBD drafted the response. Editorial comments and significant changes were submitted by the remaining authors.
